# Dengue Incidence in Urban and Rural Cambodia: Results from Population-Based Active Fever Surveillance, 2006–2008

**DOI:** 10.1371/journal.pntd.0000903

**Published:** 2010-11-30

**Authors:** Sirenda Vong, Virak Khieu, Olivier Glass, Sowath Ly, Veasna Duong, Rekol Huy, Chantha Ngan, Ole Wichmann, G. William Letson, Harold S. Margolis, Philippe Buchy

**Affiliations:** 1 Institut Pasteur–Cambodia, Phnom Penh, Cambodia; 2 National Dengue Control Program, National Center of Parasitology, Entomology and Malaria Control, Ministry of Health, Phnom Penh, Cambodia; 3 Pediatric Dengue Vaccine Initiative, International Vaccine Institute, Seoul, Korea; University of California, United States of America

## Abstract

**Background:**

Dengue vaccines are now in late-stage development, and evaluation and robust estimates of dengue disease burden are needed to facilitate further development and introduction. In Cambodia, the national dengue case-definition only allows reporting of children less than 16 years of age, and little is known about dengue burden in rural areas and among older persons. To estimate the true burden of dengue in the largest province of Cambodia, Kampong Cham, we conducted community-based active dengue fever surveillance among the 0-to-19–year age group in rural villages and urban areas during 2006–2008.

**Methods and Findings:**

Active surveillance for febrile illness was conducted in 32 villages and 10 urban areas by mothers trained to use digital thermometers combined with weekly home visits to identify persons with fever. An investigation team visited families with febrile persons to obtain informed consent for participation in the follow-up study, which included collection of personal data and blood specimens. Dengue-related febrile illness was defined using molecular and serological testing of paired acute and convalescent blood samples. Over the three years of surveillance, 6,121 fever episodes were identified with 736 laboratory-confirmed dengue virus (DENV) infections for incidences of 13.4–57.8/1,000 person-seasons. Average incidence was highest among children less than 7 years of age (41.1/1,000 person-seasons) and lowest among the 16-to-19–year age group (11.3/1,000 person-seasons). The distribution of dengue was highly focal, with incidence rates in villages and urban areas ranging from 1.5–211.5/1,000 person-seasons (median 36.5). During a DENV-3 outbreak in 2007, rural areas were affected more than urban areas (incidence 71 vs. 17/1,000 person-seasons, p<0.001).

**Conclusion:**

The large-scale active surveillance study for dengue fever in Cambodia found a higher disease incidence than reported to the national surveillance system, particularly in preschool children and that disease incidence was high in both rural and urban areas. It also confirmed the previously observed focal nature of dengue virus transmission.

## Author Summary

Dengue is a major public health problem in South-East Asia. Several dengue vaccine candidates are now in late-stage development and are being evaluated in clinical trials. Accurate estimates of true dengue disease burden will become an important factor in the public-health decision-making process for endemic countries once safe and effective vaccines become available. However, estimates of the true disease incidence are difficult to make, because national surveillance systems suffer from disease under-recognition and reporting. Dengue is mainly reported among children, and in some countries, such as Cambodia, the national case definition only includes hospitalized children. This study used active, community-based surveillance of febrile illness coupled with laboratory testing for DENV infection to identify cases of dengue fever in rural and urban populations. We found a high burden of dengue in young children and late adolescents in both rural and urban communities at a magnitude greater than previously described. The study also confirmed the previously observed focal nature of dengue virus transmission.

## Introduction

Dengue remains a major public health problem in tropical and sub-tropical countries despite aggressive efforts to control the mosquito vector [1]–[3]. Every year, an estimated 50–100 million cases of dengue fever occur and 250,000 to 500,000 cases of the more severe form, dengue hemorrhagic fever (DHF), are reported depending on epidemic activity [4]. However, the true incidence of dengue fever is not known in most disease endemic countries. Several dengue vaccine candidates have been developed and are currently being tested in clinical trials [5], [6]. Accurate estimates of dengue disease burden based on robust estimates of disease incidence will become an important factor in the public-health decision making process for endemic countries regarding the use of a safe and effective vaccine [7], [8].

In Cambodia (estimated population: 14.4 million), dengue is highly endemic and affects mainly children [9]. However, due to limited resources, the National Dengue Control Program (NDCP), which manages the national dengue reporting system, only accepts reports of clinically diagnosed cases who are <16 years of age and have been hospitalized [9]–[11]. As a result, national incidence data is thought to substantially underestimate the actual incidence of dengue since most patients are not hospitalized [11], [12]. Secondly, inherent to the NDCP case definition, the burden of dengue in late adolescence is unknown.

Historically, dengue has been considered a predominantly urban disease of tropical countries [1], probably because many early studies of dengue epidemiology were performed in urban settings and observations that annual increases in mild and severe disease incidence usually emanate from urban centers [1], [13]–[15]. However, while a high incidence of disease has been reported from rural and urban areas [16]–[18], to our knowledge no studies have compared dengue incidence in contiguous rural and urban settings.

Cambodia's national data have indicated high dengue incidences in both rural and urban districts [9]. To make a robust estimate of the actual incidence of symptomatic dengue virus (DENV) infection (i.e. dengue disease burden) in children and adolescents living in rural and urban areas of Cambodia and to describe other aspects of the epidemiology of dengue, we conducted active, community-based fever surveillance combined with diagnostic testing for DENV infection in the largest province in Cambodia during 2006–2008.

## Methods

### Surveillance population

**Figure 1:**
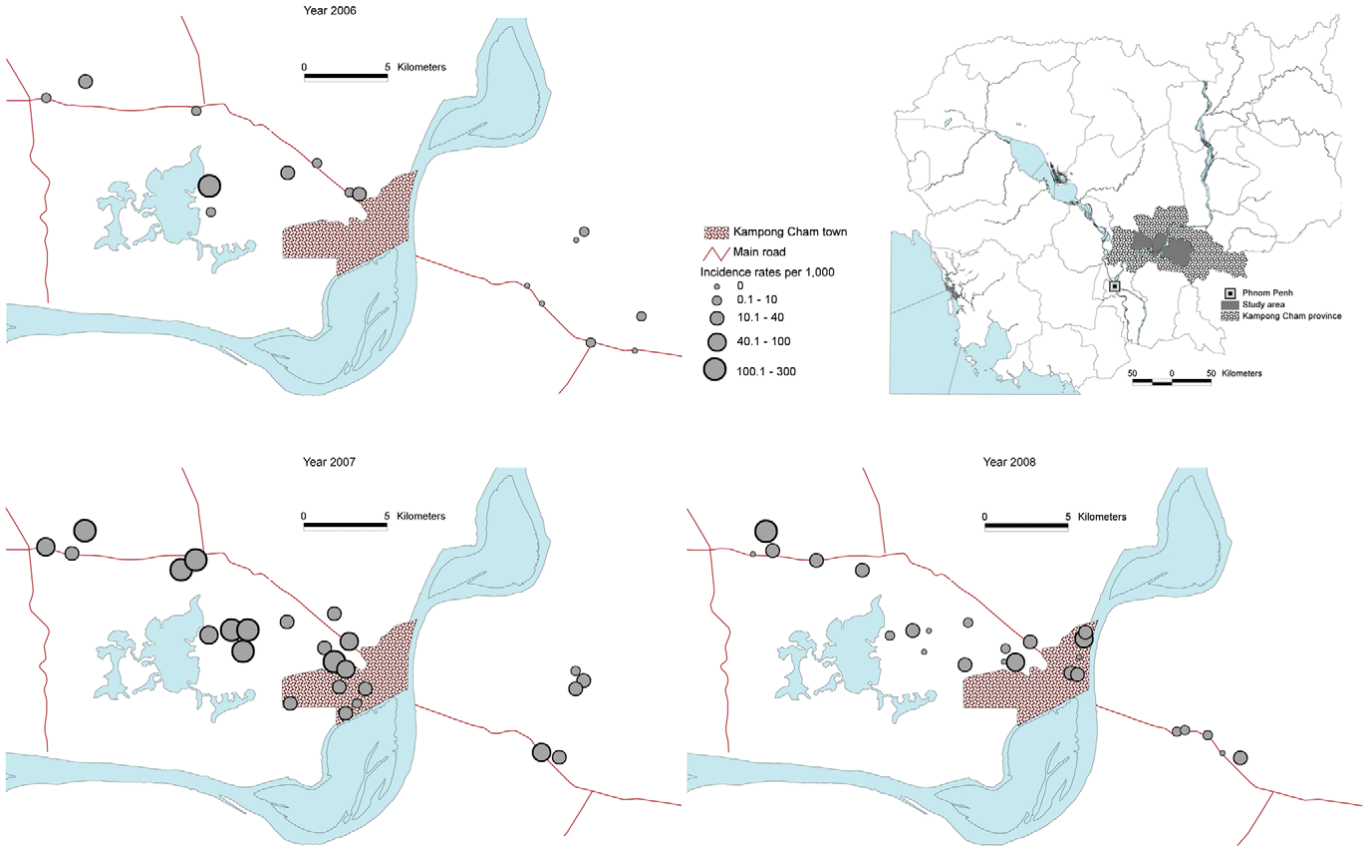
Study sites and dengue incidences by site and year, Kampong Cham province, Cambodia, 2006–2008.

Active, community-based fever surveillance was conducted in Kampong Cham (KC) province from 2006–2008 mostly during May–November, which is the rainy season and associated with increased vector activity. KC has the largest population in Cambodia and is located 120–180 km east of Phnom Penh. The provincial capital, KC town, has a population of approximately 90,000 (**Figure 1**) and a provincial hospital which has served as a sentinel site for NDCP dengue surveillance. We selected a convenience sample of villages from two rural districts located within ∼60 km radius of KC town and three districts within the KC town urban area. The population density within the urban area was estimated at ∼1900 persons/km^2^ and ∼450 persons/km^2^ within the rural villages.

### Surveillance design

Active surveillance was conducted by village teams (VT) comprised of 2–3 volunteers from each respective village and five investigation teams (IT), each comprised of a pediatric phlebotomist and an interviewer from the staff of the KC provincial hospital. Members of the VT were on the village council and selected with agreement of the respective village chiefs. VTs made weekly home visits to identify persons with fever or history of fever defined as an axillary temperature of ≥37.5°C in the previous seven days. Each VT member was responsible for a village zone and recorded home visits and temperatures in a census logbook. A team from Institut Pasteur - Cambodia (IPC) monitored VT performance on a weekly basis through unscheduled visits and frequent telephone calls. When notified by the VT about a febrile person, the IT visited the household to obtain written informed consents to participate in the follow-up part of the study from the patient or from one of the parents for children <18 years of age; children aged 12–17 years were also required to give verbal assent. A first (acute) whole blood specimen was collected within seven days of fever onset and a second (convalescent) sample was obtained on day 10–14 after fever onset. Using a standardized case report form (CRF), we collected data on demographics, illness related symptoms, type of care sought for the fever episode and duration of fever. Blood specimens were brought to KC Hospital at 4°C in insulated boxes, separated into serum aliquots, which were stored in liquid nitrogen and transported to IPC in Phnom Penh twice weekly for subsequent serologic and molecular testing.

Over the three-year period, surveillance methods were modified to expand the study. In 2006, we only enrolled subjects <16 years of age in 16 villages with documented fever ≥48 h measured using mothers' or VT's thermometers, In 2007 and 2008, surveillance was expanded to include persons <20 years of age, and a digital thermometer and a notebook to record temperatures was provided to each household along with training on how to measure and record axillary temperatures. For 2007–2008, fevers recorded by the mother regardless of duration were reported to the VT for study enrollment. During the entire study period, the IT provided the mother or patient with one day treatment of paracetamol and advice to seek medical care, but did not otherwise influence health seeking behaviors.

Participation of the village in fever surveillance was an independent decision of the respective village chiefs following a formal presentation by the study investigators. Village chiefs gave oral consent for village participation which in Cambodia is required before villagers are allowed to consent individually. The study protocol was annually reviewed and approved by the National Ethics Committee for Health Research, Phnom Penh, Cambodia and the Institutional Review Board of the International Vaccine Institute, Seoul, Korea. Relevant attributes of our observational study were reviewed using the STROBE checklist (Checklist S1).

#### Case definition

A dengue case was defined as a febrile person positive for anti-DENV IgM in the convalescent phase serum. Subjects who tested positive for anti-DENV IgM on more than one occasion during any year were only counted as one dengue case if the second febrile episode and positive test occurred within three months of the first positive episode. However, if the second febrile, IgM anti-DENV positive episode was also DENV positive by RT-PCR, that was classified as a second case of dengue in the same person.

### Laboratory testing

All acute and convalescent serum specimens were tested for both anti-DENV IgM and anti-Japanese Encephalitis virus (JEV) IgM using in-house capture enzyme-linked immunosorbent assays (MAC-ELISA) as described by Rossi and Ksiazek [19] and adapted for DENV and JEV diagnosis [20]. Briefly 96-well microtiter plates were coated with goat anti-human IgM and 100 µL of serum samples and controls diluted 1∶100 in phosphate buffered saline + Tween 20 0.5% (PBS-T) +5% nonfat dried milk (PBS-T-NDM) were added and incubated for one hour at 37°C. After washing with PBS-T, DENV (DENV-1 Hawaii, DENV-2 New Guinea, DENV-3 H-87, DENV-4 H-241) or JEV (Nakayama) suckling mouse-brain derived antigen produced by sucrose acetone extraction [21] was added to each well at a concentration of 16 hemagglutination units, and incubated at 4°C overnight. The plate was washed with PBS-T, monoclonal antibody to the antigens being detected was added, incubated at 37°C, washed and peroxidase conjugated anti-mouse Fab H+L was added. The final reaction was developed and read at 405 nm on an automated plate reader. A result was considered positive when the optical density (OD) was > mean OD of the three negative control specimens + three standard deviations. When the anti-JEV result was higher than the anti-DENV result the subject was not considered to have DENV infection.

Only acute phase sera of participants who were positive for anti-DENV IgM in the convalescent sample were tested for DENV using molecular methods. DENV ribonucleic acid amplification, detection and serotyping were performed using reverse transcriptase polymerase chain reaction (RT-PCR) according to *Lanciotti* [22] as modified by *Reynes et al* [23]. Briefly, RNA was extracted from 140 µL of serum (QIAamp Viral RNA Mini kit, QIAGEN) or from 200 µL of specimen (MagNA Pure Compact Nucleic Acid Isolation Kit I, Roche Diagnostic) on a MagNA Pure Compact instrument. First round RT-PCR was carried out in a 25-µl volume containing 2.5 µl of extracted nucleic acid, 0.2 mM (each) dNTPs, 5 X *Taq* buffer, 1 µl of Qiagen One-step RT-PCR kit, 0.2 µM each D1 and D2 outer primers [24], and PCR-grade water with thermal-cycler (MyCycler, Bio-Rad) settings of 60 min of reverse-transcription at 45°C, 2 min denaturation at 92°C followed by 35 cycles consisting of 94°C for 30 sec, 55°C for 30 sec and 72°C for 1 min, and a 72°C final extension step for 10 min. Semi-nested PCR was performed in a 50-µl reaction mixture containing 2.5 mM MgCl_2_, 0.2 mM (each) dNTPs, 10X *Taq* buffer, 1.2 U of *Taq* polymerase, 5 µl of diluted first-round PCR product diluted 1∶100 and 0.2 µM (each) D1, TSI-modified, TS2, TS3 and TS4 primers [23]. The cycling program consisted of a 5-min denaturation step at 94°C, followed by 25 cycles each at 94°C for 30 sec, 55°C for 30 sec, and 72°C for 1 min, and ended with 72°C extension for 10 min. Positive and negative controls were included for each step of the process. Detection was by agarose gel electrophoresis for the expected amplicon sizes.

Dengue virus isolation was attempted on all PCR positive specimens using C6/36 (*Aedes albopictus*) and Vero E6 cells, with detection by direct fluorescent microscopy with serotype-specific monoclonal antibodies as previously described [24].

### Data analysis

Proportions, rates, risk ratios (RR), odds ratios (OR), 95% confidence intervals (CI) and statistical tests were calculated using STATA version 9.2 (StataCorp, College Station, TX, USA). Dengue incidences for the study periods, which encompassed the dengue season, were not annualized; we assumed they represented the annual incidence because of the marked seasonality of dengue. We calculated incidence rates using person-weeks to illustrate time series, accounting for the actual number of persons under surveillance per week. In addition, to account for the length of time during which the participants were followed up by age group, we calculated age-specific incidence rates using person-season. One person-season corresponded to one participant followed during the entire surveillance period of the respective year. A child was declared lost to follow-up when he/she was not present during 3 weekly home visits in a row. Logistic regression models were used with stepwise forward variable selection to identify independent predictors with p-to-enter of 0.10 or less.

## Results

### Characteristics of the surveillance population

Over the three years, 32 villages and 10 urban areas participated in active surveillance for febrile illnesses; five villages for three years, 13 for two years and 14 for one year. Thirty-four percent of the population of these villages or urban areas were covered by the surveillance study and ranged from 6.2–78.6% per village or urban area. A total of 6,657 children aged <16 years from 16 villages were in the surveillance study during May 08-November 23, 2006, while 10,086 and 7,673 individuals <20 years of age were under surveillance during June 01-December 31, 2007 and April 01 to December 31, 2008, respectively (**Table 1**). A total of 2,575 (25.5%) surveillance participants resided in urban areas in 2007 and 1,024 (13.2%) in 2008. Over the study period, 52% were males and the median age of the participants was 6 years among the 0–15 year age group. No differences in gender distribution were noted between years. The number of refusals to participate in the study was 0.6% per year on average (range 0.4–0.9%). Few participants (0.9% on average; range 0.8–1.1%) moved outside the surveillance area (**Table 1**).

**Table 1:** Characteristics of participants in febrile illness surveillance study, Kampong Cham province, Cambodia, 2006–2008.

Year		2006	2007	2008
**Rural villages & Urban areas (number)**		16 & 0	20 & 5	20 & 5
**Percentage of village populations covered by active surveillance**				
Median coverage		34.8%	36.4%	32.9%
Min-Max		12.8–69.8%	6.2–78.6%	11.0–48.4%
**Active Fever surveillance**				
Surveillance period in days (in months)		199 (6)	214 (7)	275 (8)
No of participants (% urban population)		6,657 (0)	10,086 (25.5)	7,673 (13.2)
Median days of surveillance for each participant		195	210	259
Minimum - maximum (min - max) of surveillance		8–199	14–214	14–275
Number of refusals to participate (%)		37 (0.6)	44 (0.4)	68 (0.9)
Number of participants lost to follow-up (%)		76 (1.1)	91(0.9)	59 (0.8)
**Males (%)**		51.9	51.9	50.5
**Age group distribution (%)**				
0–4 years		25.3	22.9	23.4
5–9 years		31.4	25.6	26.4
10–14 years		43.0*	29.2	29.5
15–19 years		NA	22.4	20.8
Mean		8.1	9.5	9.4
Median (min–max)		8 (0–15)	10 (0–20)	10 (0–20)
**Number of febrile episodes**		1,633	3,088	2,851
**Number of febrile episodes with blood samples (number of individuals)**		444 (426)	2,953 (2,474)	2,724 (2,095)
- **Fever events per person**				
Mean		1.15	1.22	1.34
Median (min–max)		1 (1–5)	1 (1–5)	1 (1–6)
**Incidence rates of febrile illness per 1,000 person-seasons**				
**- Overall**		244.0	336.9	429.5
**- Urban areas**		NA	176.1	164.9
**- Rural areas**		244.0	390.0	470.8
**- By village**				
- Number of villages/areas with fever episodes		16	25	25
- Mean		196.5	381.4	436.8
- Median		193.0	315.0	414.8
- Min–max		31.5–368.1	137.5–1002.2	39.0–1018.1

*data pertaining to 10–15 year age group in 2006; NA: not available.

### Characteristics of non-dengue and dengue cases

Over the study period, 6,121 febrile episodes were reported from 4,995 individuals who were bled and tested for DENV infection (data by year shown in **Table 1**). Of these febrile episodes, 736 (12%) had a laboratory-confirmed DENV-infection with 425 (58%) being confirmed by RT-PCR (**Table 2**). DENV was isolated by cell culture in 292 (69%) of PCR-positive samples. No dengue-related deaths were reported among the study population. The median age of dengue cases was 7 years and 51% were males. In 2006 and 2008, 89 and 117 dengue cases were detected yielding an incidence of 13.4/1,000 and 17.6/1,000 person-seasons, respectively. In 2007, a large epidemic occurred with 530 dengue cases resulting in an incidence of 57.8/1,000 person-seasons. Over the three years, the majority of cases occurred during May–August (86% on average). Only 3.9% of dengue cases reported fever that lasted <2 days. Significant differences in the proportions of cases requiring hospitalization were noted between years: 41.6%, 10.6% and 2.6% in 2006, 2007 and 2008 respectively (p<0.001) (**Table 2**). Over the three years, among febrile patients, those with DENV-infection sought care more often (52.7% versus 23.8%, p = <0.001) and were more frequently hospitalized (RR 8.0; 13.6% vs. 1.6% p<0.001) than those without DENV. Among persons with febrile illness, the duration of fever was significantly longer among those with DENV infection compared to those without DENV infection (4.4 days vs. 3.7 days, p<0.001) (**Table 3**). The duration of fever between the 0–9, 10–14 and 15–19 year-age groups was not significantly different (4.5, 4.2 and 4.3 days respectively, p values>0.16). Similarly, hospitalization rates among persons with dengue were not significantly different between the 3 age-groups (29.3%, 9.3% and 23.8% respectively, p values>0.15).

**Table 2:** Characteristics of dengue virus infected febrile patients, surveillance study villages, Kampong Cham province, Cambodia 2006–2008.

Year		2006	2007	2008
**Number of dengue cases**		89	530	117
**Age in years**				
Mean		6.6	7.1	8.3
Median		6	6	8
Min–Max		0–15	0–19	1–17
**Duration of fever** **Number of cases (%)**				
Fever <4		15 (16.9)	189 (35.7)	45 (38.5)
Fever = 4		14 (15.7)	110 (20.8)	35 (29.9)
Fever >4		60 (67.4)	231 (43.6)	37 (31.6)
**Patient outcomes** **Number of cases (%)** *				
Hospitalized		41 (46.1)	56 (10.6)	3 (2.6)
Outpatient care		45 (50.5)	222 (41.9)	36 (30.8)
Did not seek care		3 (3.4)	252 (47.5)	78 (66.7)
**Laboratory results:** **Number (%)**				
Positive by RT-PCR		32 (35.9)	320 (60.4)	72 (61.5)
serum collected 0 – 4 days of fever onset		20/28 (71.4)†	217/262 (82.8)	56/65 (86.2)
serum collected >4 days of fever onset		12/61 (19.7)	103/268 (38.4)	16/52 (30.1)
Positive virus isolation		21 (23.6)	214 (40.4)	57 (48.9)
**Dengue serotypes** **Number (%)**				
DENV-1		25 (78.1)	90 (28.1)	0
DENV-2		2 (6.3)	1 (0.3)	56 (77.5)
DENV-3		5 (15.6)	217 (67.8)	12 (16.9)
DENV-4		0	12 (3.8)	4 (5.6)
**Incidence rates of dengue fever per 1,000 person – seasons**				
**- Overall**		13.4	57.8	17.6
**- By age group (years)**				
0–4		18.0	81.7	13.2
5–9		15.9	84.2	19.0
10–14		8.1**	40.2	13.7
15–19		NA***	12.6	4.7
**- Urban areas**		NA	17.1	21.2
**- Rural areas**		13.4	71.3	17.1
**Dengue cases by village or urban area**				
- Number with ≥1 dengue case (%)		12 (75)	25 (100)	21 (84)
- Mean		16.2	68.2	25.1
- Median (min–max)		6.5(1.5–108.1)	36.5(5.6–211.5)	16.9(3.3–127.4)

*not mutually exclusive;

**data only available for <16 year age group in 2006;

***NA: not available;

†serum collected 2–4 days of fever onset in 2006.

**Table 3:** Variables associated dengue virus infection among persons with febrile illness compared with non-dengue febrile illness, Kampong Cham province, Cambodia, 2006–2008.

	TOTAL		DENGUE		Non DENGUE		P value	RR**	95%CI**
	n = 6121	%	n = 736	%	n = 5385	%			
**Age in years**									
Mean	7.1	-	7.2	-	7	-	0.651		
Median	7	-	7	-	7	-			
Min–Max	0–19	-	0–19	-	0–19	-			
**Gender**									
Males	3,132	51.4	372	53.6	2,760	53.1	0.767		
**Duration of fever in days**									
Mean	3.80	-	4.41	-	3.71	-	**<0.001**		
Median	4	-	4	-	4	-			
Min–Max	0–20	-	0–18	-	0–20	-			
**Fever resulting in care seeking**	1,701	27.2	388	52.7	1,313	23.8	**<0.001**	2.1	1.9–2.4
**Care at:**									
Pharmacy	321	5.1	34	4.6	287	5.2	0.405	0.9	0.6–1.2
Health center	191	3.1	27	3.7	164	3.0	0.372	1.2	0.8–1.8
Private clinic	997	16.3	236	32.1	761	13.8	**<0.001**	2.2	1.9–2.6
Hospitalization	191	3.1	106	13.6	91	1.6	**<0.001**	8.0	6.0–10.8

**RR  =  risk ratio; 95% CI  =  confidence interval.

### DENV transmission pattern

**Figure 2:**
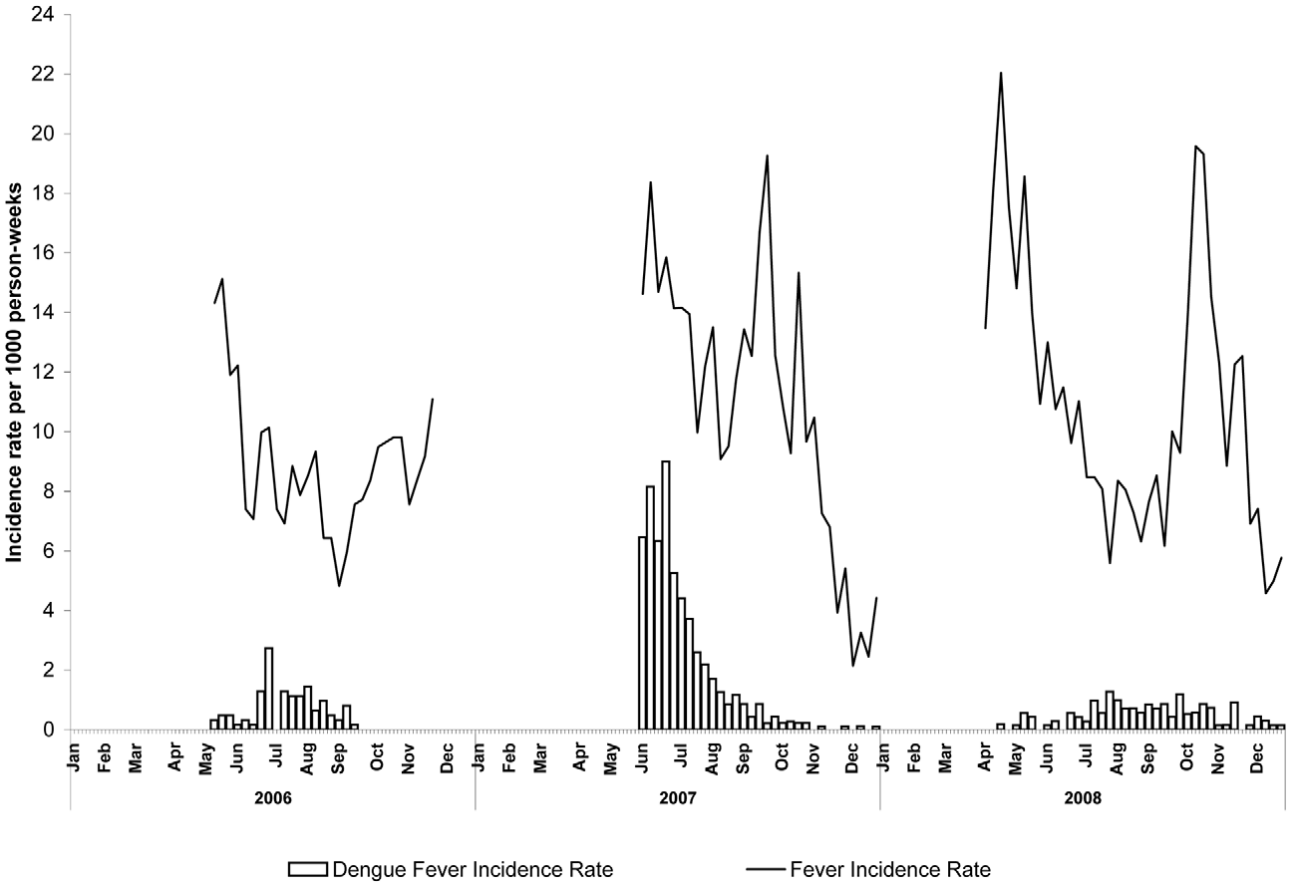
Incidence of Febrile Illness and Dengue Fever by Week, Kampong Cham Province, Cambodia, 2006–2008.

In 2006 and 2008, dengue cases occurred in 12 (75%) and 19 (76%) of villages respectively (among affected villages, median 1 case per village in both years). The patterns of dengue by village varied considerably from year to year. During the 2007 epidemic, all villages were affected by dengue: median incidence rate by village was 36.5/1,000 person-seasons (range by village 5.6–211.5/1,000) including eight (32%) villages with incidence rates ≥100/1,000 person-seasons. Highest incidence rates by village observed in 2008 and 2006 were 127.4/1,000 and 108.1/1,000 person-seasons respectively. The incidence in rural areas in 2007 was 4.2-times higher than that in urban areas (71.3 versus 17.1/1,000 person-seasons, p<0.001). The following year, the relative risk reversed. The risk ratio was 0.81 when rural with urban areas (17.1/1,000 vs. 21.2/1,000 person-seasons; p = 0.01) (**Figure 2**).

**Figure 3:**
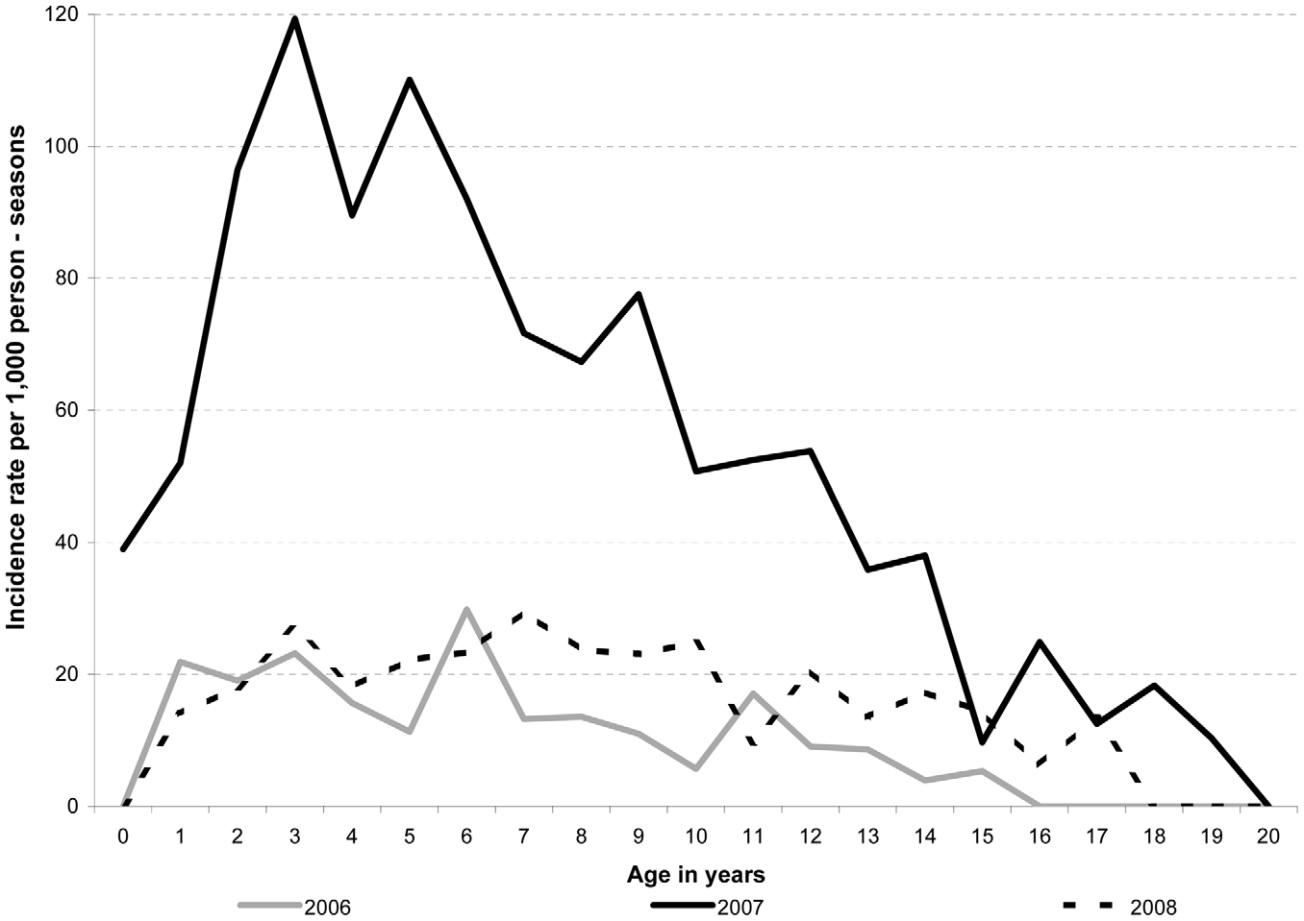
Dengue Incidence Rates by Age, Kampong Cham Province, Cambodia, 2006–2008.

Highest age-specific incidence rates were observed among the 0–4 and 5–9 year age-groups and tended to be 2–3 times higher than that of the 15–19 year-olds (**Table 2**). Peak ages of dengue by year are shown in Figure 3 with children aged 3, 6, and 7 comprising the highest incidence in 2006, 2007, and 2008, respectively.

### DENV-serotype distribution and associations with frequency of hospitalization

All four serotypes were identified over the study period, but DENV-3 (n = 234, 55.3%) and DENV-1 (n = 115, 27.2%) accounted for most (**Table 4**). In 2006, DENV-1 accounted for 78% of the cases that were PCR positive (25/32), was mainly found in one village (18 of 19 PCR positive cases) and was associated with a large outbreak (59 cases; incidence rate 108.1/1,000 person-seasons during 11 weeks). Of these 18 DENV-1 cases in the village, 8 (44.4%) were hospitalized and significantly younger than non-hospitalized children with DENV infection (mean age 5.3 [age range 0.3–12] versus 7.3 years [age range 0.8–15] respectively, p = 0.043). DENV-3 (67.8% of cases) was associated with the 2007 epidemic and was replaced by DENV-2 (77.5% of cases) in 2008 (**Table 2**). Co-infection with DENV-1 and -3 was observed in 2 patients. Patients with DENV-4 (n = 16) were rare and of these, 8 (50%) patients resided in one village in 2007.

**Table 4:** Characteristics associated with serotypes, Kampong Cham province, Cambodia, 2006–2008.

	DENV-1	DENV-2	DENV-3	DENV-4	All confirmed cases	DENV-1 vs. -2		DENV-1 vs. -3		DENV-2 vs. -3	
	n = 115	n = 58	n = 234	n = 16	n = 736	aOR**	p value	aOR	p value	aOR	p value
**Duration of fever in days:**											
<4	31.3	43.1	28.2	56.3	18.5	ref.*		ref.		ref.	
= 4	14.8	31.0	23.1	18.8	12.5	1.7	0.279	1.8	0.105	1.0	0.935
>4	53.9	25.9	48.7	25.0	26.5	0.4	**0.050**	1.2	0.588	2.7	**0.011**
**Patient outcomes (%)**											
Hospitalized	7.8	0.0	14.5	12.4	10.8			3.1	**0.045**		
Non-inpatient care∧	54.8	41.4	38.6	31.3	42.8						
Did not seek care	37.4	58.6	46.9	56.3	42.4						
**Age distribution in years (%)**											
0–4	30.4	25.9	37.2	18.8	33.1	ref.		ref.		ref.	
5–9	41.7	36.2	41.5	50.0	41.1	1.0	0.982	0.8	0.508	0.8	0.522
10–14	24.3	34.5	17.1	18.8	21.5	1.9	0.187	0.7	0.299	0.4	0.009
≥15	3.5	3.4	4.3	12.5†	4.3	1.7	0.512	1.0	0.980	1.1	0.935

*ref.  =  reference group;

**aOR  =  adjusted Odds Ratio using logistic regression model, which included *age*, *fever duration* and *patient outcomes* as independent variables.

∧ pharmacy, outpatient clinic, etc.

**†:** DENV-4 was not significantly more frequent in the 15–19 age group compared with other serotypes (p>0.30).

DENV-1 or DENV-3 infection presented with fever duration that was longer when compared to DENV-2: 53.9% of DENV-1 and 48.7% of DENV-3 infected subjects had fevers lasting >4 days versus 25.9% of DENV-2 (p values≤0.05, **Table 4**). Interestingly, of DENV-2 infected patients none was admitted to hospital compared to 11.3–14.5% for DENV-1, -3 or -4. Multivariate analyses performed comparing DENV-1 and -2; DENV-1 and DENV-3, and DENV-2 and -3 retained an independent association between DENV-2 and shorter fever duration when compared with DENV-1 (p = 0.050) and -3 (p = 0.011). Furthermore, DENV-2 infection was more often observed in older children when compared to DENV-3 infection (p = 0.009, **Table 4**). Hospitalization was more frequently associated with DENV-3 infections compared with DENV-1 (11.3% vs. 14.5%; adjusted OR 3.1; p = 0.045) (**Table 4**). Of note, when considering only year 2007, DENV-3 infection was also associated with higher frequency of hospitalization when compared with DENV-1 (15.7% (34/216) vs. 4.4% (4/90), p = 0.006).

## Discussion

Assessments of transmission patterns and true incidences of dengue or DENV-infections are resource intensive and have been performed in a limited number of settings. Multi-year studies with active, community-based surveillance for febrile illness have been conducted in South East Asia (Kamphaeng Phet [25] and Ratchaburi, Thailand [26], A Sabchareon, personal communication, unpublished data); Long Xuen, Viet Nam [27] and Bandung, West Java, Indonesia [28] and in the Americas (Iquitos, Peru [29] and Managua, Nicaragua [30], [31]). Only one study determined the incidence of dengue among adults (18–66 year-old factory workers) in Indonesia [28]. All the others determined disease incidence in children using various active surveillance methods to detect febrile illness, including school absenteeism and self-reporting by participants. All studies included clinical follow-up to identify patients for serologic and molecular testing for DENV infection. Similar to our study, surveillance was mainly conducted during the dengue season in Kamphaeng Phet Thailand), while sites in Ratchaburi, Thailand, Vietnam, Nicaragua, Peru and Indonesia conducted year-round surveillance. However, all these studies only conducted surveillance in relatively populated urban or semi-urban areas. In addition, community-based active surveillance for febrile illness in all age groups was conducted during several large dengue epidemics in rural and urban areas of Cuba in 1997 and 2001 [32], [33] although only surveillance data from urban areas were published.

Although there were some methodological differences in how active surveillance was conducted in our study and those identified above (e.g., house-to-house visits compared to school absenteeism or self-identification of fever episodes), we found annual disease incidence rates of 1.3–5.7% over three years, which was higher than those observed in Kamphaeng Phet (0.8–3.6% in 4–14 year-olds) or Ratchaburi, Thailand (1.6% in 4–14 year-olds) (26), Nicaragua (0.4–1.9% in 2–11 year-olds), Vietnam (1.7–4.0% among 2–15 year-olds) or Peru (1.0% in 5–17 year-olds). Moreover our highest incidence rates were among pre-school children ages compared to Thailand and the Americas where the highest age-specific incidence rates were among children >8–9 years of age [25], [31].

For several reasons the incidence observed in our study may still have underestimated the true disease burden although our use of house-to-house case finding was most likely to identify febrile children in a low-literacy rate population where attendance at public health centers is thought to be low [34], [35]. First, surveillance was not performed throughout the year and dengue cases are known to occur at low rates between dengue seasons. However, based on nationally reported cases, those that occur during the non-dengue season only account for <5% of the total [9]. Second, in the first year of the study (2006) our case definition required a fever ≥48 hrs and only a third of children with a febrile episode met those criteria. However it is unlikely that a significant number of cases were excluded since in subsequent years <4% of dengue cases had fever duration <2 days. Third, in 2007 a large dengue epidemic occurred in the country resulting in ∼4 fold increase of cases reported to NDCP as compared to the previous 5 years [9]. Since the NDCP data indicated that the epidemic in 2007 started in Cambodia approximately one month before our surveillance began and accounted for ∼25% of the total reported cases [9]; we may have missed this proportion of cases prior to our start of active fever surveillance in June. Finally, our laboratory diagnostic algorithm only tested acute phase samples from patients with fever when the convalescent sample tested positive for anti-DENV IgM. In a setting such as ours where there is a high frequency of secondary DENV infections, up to 28% of secondary infections might not be detected serologically because of low IgM levels [36].

Dengue is highly focal in its geographic distribution and this was observed during this surveillance study. Dengue incidences by village showed wide ranges and a markedly diverse spatial distribution within the study area and population. DENV-3 predominated in 2006 in Cambodia and was reported in 16 of 24 provinces in Cambodia (Ministry of Health's unpublished data). However, during the same year an outbreak in one of the participating villages was caused by DENV-1 and was responsible for two-thirds of all identified cases in the study area. In 2008, one village was affected by DENV-2 with an attack rate of ∼13% and accounted for 34% of all identified cases. A wide range of incidences among the villages and urban areas was also observed during the 2007-epidemic.

Another finding in this surveillance study was that rural areas were usually affected by dengue to the same degree as urban areas or, as during the 2007-epidemic, at even higher attack rates. Factors thought to explain the perceived urban predominance of dengue included rapid human population growth, the presence of an unreliable or absent water supply which forced residents to store water in open containers and the presence of other man-made containers, all of which serve as major production sites for *Aedes aegypti* [35], [37], [38] All of these conditions are present in rural areas of Cambodia as the country develops without proper water supplies and persons from rural areas frequently travel between large cities and relatives in their villages. The villages in our surveillance study were located close to one of the main national roads with constant daily traffic between villages and Phnom Penh and the opportunity for dengue introduction from viremic individuals passing through the area. The expansion of dengue to rural areas has also been observed in Thailand and India [16]–[18]. In the absence of a dengue vaccine, this finding underscores the importance of conducting education and vector control programs in rural as well as urban areas of Cambodia. The high dengue incidence in children <7 years of age who rarely leave the villages and are not yet in school, argues for ongoing transmission within Cambodian villages and around households as has been recently observed in Thailand [39].

Assuming that hospitalization was a surrogate for disease severity, 2006 appeared to have been particularly severe with 46% of dengue cases hospitalized. However, our study area may not have been representative of the country. A DENV-1-related outbreak occurred primarily in one village in the study area and resulted in a higher hospitalization frequency among younger children, while the predominant virus circulating in the country was DENV-3. Because DENV-1 has circulated less frequently than DENV-2 or DENV-3 in Cambodia [9], it may have caused more severe disease expression among a largely susceptible population. In subsequent years, DENV-4 and DENV-2 appeared to have been associated with milder disease than DENV -1 and DENV-3. Less severe disease expression has been previously described for DENV-4, however, milder expression of DENV-2 dengue came as a surprise since this serotype was associated in previous studies from Thailand with increased disease severity [40], [41]. Nevertheless, clinical expression may also depend on genetic sub-strains within the four serotypes, pre-existing antibody to DENV or the sequence of infections with other serotypes [42]. This phenomenon of apparent attenuation of dengue disease severity at a population level was also seen observed after the large epidemics of 1998 in the Philippines and Thailand [43], [44]. We were limited in further analyses since circulation of serotypes was year-dependent: only 5% of DENV-2 detected in this study circulated in 2006 and 2007 and no DENV-1 was identified in 2008.

Our surveillance study highlights once again the high degree of under-recognition and under-reporting of this disease and the variation in dengue epidemiology which occurs annually because of the cyclical variation in incidence. A number of factors are thought to influence DENV transmission and disease expression such as environment [45], human and vector behaviors [46], possible virulence of the virus strains [13], [42], [43], and the human host [42], [47], [48] (baseline immunity and cross-protective immunity). Given the cyclical nature of dengue incidence with broad variations from one year to another, we underscore the importance of maintaining long term population-based surveillance sites to better estimate true incidence and define the dynamics of dengue epidemiology and serotype circulation. Long-term epidemiological data on dengue and DENV transmission obtained though population-based surveillance studies will also be needed to for evaluation of promising dengue vaccine candidates both through randomized, controlled clinical trials of efficacy [49] and studies of vaccine effectiveness.

## Supporting Information

Checklist S1 STROBE Checklist
(0.09 MB DOC)
